# Pregnancy after kidney transplantation: an observational study on maternal, graft and offspring outcomes in view of current literature

**DOI:** 10.3389/fneph.2023.1216762

**Published:** 2023-07-27

**Authors:** Louis Stavart, Camille Verly, Jean-Pierre Venetz, David Baud, Helene Legardeur, Yvan Vial, Dela Golshayan

**Affiliations:** ^1^ Transplantation Center, Lausanne University Hospital (CHUV), Lausanne, Switzerland; ^2^ Faculty of Biology and Medicine, University of Lausanne (UNIL), Lausanne, Switzerland; ^3^ Service of Gynecology and Obstetrics, Woman-Mother-Child Department, Lausanne University Hospital (CHUV), Lausanne, Switzerland

**Keywords:** pregnancy, kidney transplantation, immunosuppressive drugs, graft survival, preeclampsia, offspring, maternal-fetal medicine

## Abstract

**Introduction:**

Pregnancy after kidney transplantation (KTx) is considered to have a high risk of non-negligible complications for the mother, the allograft, and the offspring. With an increased incidence of these pregnancies over the past decades, transplant nephrologists and specialized obstetricians face increasing challenges, with scarce literature regarding long-term outcomes.

**Methods:**

We retrospectively collected data from all women with at least one live birth pregnancy after KTx who were followed at our tertiary hospital between 2000 and 2021 to study maternal, graft and fetal outcomes.

**Results:**

Ten patients underwent 14 live birth pregnancies after KTx. Preponderant maternal complications were stage 1 acute kidney injury (43%), urinary tract infections (UTI, 43%), progression of proteinuria without diagnostic criteria for preeclampsia (29%), and preeclampsia (14%). Median baseline serum creatinine at conception was 126.5 µmol/L [median estimated glomerular filtration rate (eGFR) 49 mL/min/1.73m^2^], and eGFR tended to be lower than baseline at follow-ups. Overall, there was no increase in preexisting or occurrence of *de novo* donor-specific antibodies. No graft loss was documented within the 2-year follow-up. There were nine premature births (64%), with a median gestational age of 35.7 weeks. The median birth weight, height, and head circumference were 2,560 g, 45.5 cm, and 32.1 cm, respectively. These measurements tended to improve over time, reaching a higher percentile than at birth, especially in terms of height, but on average remained under the 50th percentile curve.

**Discussion:**

Overall, pregnancies after KTx came with a range of risks for the mother, with a high prevalence of cesarean sections, emergency deliveries, UTI, and preeclampsia, and for the child, with a high proportion of prematurity, lower measurements at birth, and a tendency to stay under the 50th percentile in growth charts. The short- and long-term impact on the allograft seemed reassuring; however, there was a trend toward lower eGFR after pregnancy. With these data, we emphasize the need for a careful examination of individual risks via specialized pre-conception consultations and regular monitoring by a transplant nephrologist and a specialist in maternal–fetal medicine during pregnancy. More data about the long-term development of children are required to fully apprehend the impact of KTx on offspring.

## Introduction

Impaired fertility in women with chronic kidney disease (CKD) and end-stage kidney disease (ESKD) is attributed to many physiological changes, including altered levels of sexual hormones and psychological disturbances related to chronic illness (1). Successful kidney transplantation (KTx) thus provides an unexpected opportunity for motherhood by rapidly restoring reproductive mechanisms and, in the absence of complications, improved overall wellbeing (2–4). Since the two first reported pregnancies (in 1958 and 1960) in a 33-year-old twin-sister living-donor KTx recipient (5), dealing with pregnancies in women after transplantation has become an expanding part of clinical practice at transplantation and obstetrical centers (6, 7).

Although women who have received KTx (henceforth referred to as ‘KTx women’) tend to have greater birth rates and a better maternal outcome than women with CKD, their pregnancies are still considered high risk, with non-negligible maternal, graft, and fetal risks (1, 8). Indeed, KTx women are more susceptible to preeclampsia, more frequently develop arterial hypertension or gestational diabetes, and have a greater risk of delivery by cesarean section (9). Furthermore, pregnancy may trigger allograft dysfunction, such as new-onset proteinuria or increased serum creatinine (sCr) levels that may persist after delivery (6). Alloimmunization with the occurrence of donor-specific anti-human leukocyte antigen (HLA) antibodies (DSA) are also well-described within the setting of pregnancy (10). Interestingly, the rates of acute rejection and graft loss in pregnant KTx women do not seem to differ from those in non-pregnant KTx women (6, 8, 9). Finally, KTx women’s newborns suffer from higher rates of prematurity and from lower anthropometric measurements (9). Despite these risks, increasing numbers of KTx women with at risk profiles are contemplating pregnancy (11, 12), creating a need for a better understanding of the determinants of adverse outcomes and the implementation of preventive measures. Literature remains relatively scarce, with a focus on maternal and graft outcomes. Above all, little information exists on the long-term development of the offspring, but emerging available data is rather reassuring (13, 14). In addition, meta-analyses have revealed that outcomes tend to vary across geographical regions (8, 9). On one hand, global multi-center and detailed prospective registries are needed to improve the care of KTx women and their offspring. On the other hand, clinicians will have to weigh patient and health system-related local disparities when implementing international policies and decision algorithms in pre-counseling consultations. We thus report our experience with pregnancy after KTx through mother and child outcomes at our tertiary center, one of the six KTx centers in Switzerland.

## Methods

### Study population

We screened all women who had a KTx between January 2000 and December 2021 in our tertiary hospital (Lausanne University Hospital, Switzerland). We retrospectively included all pregnancies after KTx that were regularly followed at our Transplantation Center and resulted in at least one live birth. Indeed, due to the retrospective nature of our study, we could not obtain accurate data regarding the early termination of pregnancies or miscarriages in all KTx women with follow-up at our center. Patients were excluded if medical charts could not be retrieved. During the defined period, 943 KTx operations were performed on 626 men and 317 women. The number of women of childbearing age (15–49 years) was 143, representing 45.1% of transplanted women.

### Data collection

The collected clinical data were retrieved from medical records for all the pregnancies during the study period, which amounted to 14 live births in 10 different patients after KTx. Follow-ups for the mother and the allograft were conducted at the following time points: within 3–6 months before pregnancy; at the 1st, 2nd, and 3rd trimester of pregnancy; and at 6 months, 1 year, and 2 years after delivery.

#### Maternal baseline and pregnancy-related data

We collected the following patient data: age at conception, type of donation (living- *vs.* deceased-donor organ), time between transplantation and conception, number of transplantations before conception, diagnosis of nephropathy, preexisting comorbidities (diabetes and other endocrine diseases, and arterial hypertension), and height and weight of the mother [to calculate body mass index (BMI)]. Regarding pregnancy, we collected the following data: gravidity and parity, duration of gestation, type of birth (vaginal delivery *vs*. cesarean section), complications during pregnancy, and any peculiarity in the newborn.

#### Kidney allograft function

The following clinical and laboratory data were collected at different time points before and during pregnancy and postpartum: blood pressure (mmHg), sCr levels (µmol/L), proteinuria, immunosuppressive drugs, levels of anti-HLA antibodies and DSA, and the occurence of graft rejection episodes and biopsies. Baseline sCr, trimestral sCr, and follow-up sCr levels were defined as the mean of all available values within the 3 months before the estimated date of the last menstrual period (baseline kidney function before pregnancy), within 1–2 weeks of the relevant trimestral date, and within 1 month of the post-partum follow-up date, respectively. Urine protein/creatinine ratio (uPCR, mg/mmol), patient weight, and arterial blood pressure were computed using the same time restrictions. For relevance purposes, sCr and uPCR were considered “not applicable” if there was no available value in the mentioned follow-ups. The estimated glomerular filtration rate (eGFR) was calculated according to the 2021 Chronic Kidney Disease Epidemiology Collaboration (CKD-EPI) equation (15). Laboratory data were considered “not applicable” if the patient had undergone another KTx between time points (e.g., Patient 3). Acute kidney injury (AKI) was classified according to the sCr criteria of the Kidney Disease: Improving Global Outcomes guidelines (16). Graft loss was defined as the need for any renal replacement therapy (RRT), that is, hemodialysis, peritoneal dialysis, or preemptive KTx. Preeclampsia was defined in accordance with the American College of Obstetricians and Gynecologists criteria (17). Hypertension was defined as a systolic blood pressure > 140 mmHg and/or a diastolic blood pressure > 90 mmHg or the use of antihypertensive medication at conception (chronic hypertension) and after 20 weeks of gestation (gestational hypertension), in accordance with National Institute for Health and Care Excellence guidelines (18).

#### Newborn characteristics

The newborn’s weight, height, and head circumference measurements were extracted from the mother’s medical chart and were divided into percentiles according to the gestational growth charts validated in 2011 by the Swiss Society of Pediatrics, based on a German cohort in 2006 (19, 20). Newborns were considered small for gestational age (SGA) if their weight was under the 10th percentile. Each KTx mother was given a questionnaire to gather information, retrospectively, about previous pregnancies, pregnancies after KTx, and the long-term development of their children (**Supplementary Figure 1**). Follow-up data on the child’s weight and height were also classified according to age using the Swiss validated growth charts (20), with the last follow-up date set as the day of the completion of the questionnaire.

### Statistics

Each continuous variable is expressed as the median (range). The statistics are mainly descriptive, due to the small cohort. All computations and figures were made with R, version 4.2.0.

## Results

### Patients’ characteristics

Maternal and obstetrical characteristics are summarized in **Tables 1**, **2**, respectively. Median age at conception was 35 years (range 23–42 years). All women were caucasian, except one patient of Asian descent (i.e., Sri Lanka). Median BMI at conception was 22 kg/m^2^ (range 17-26 kg/m^2^), with no obese patients but one underweight patient. In addition, 30% of the patients were known to have chronic hypertension, which was diagnosed before pregnancy. There was no occurrence of hypertension in our patients before 20 weeks of pregnancy. Aspirin was not systematically prescribed preventively (only 1/10 patients received aspirin, i.e. Patient 3 for her third pregnancy) and one patient received therapeutic anticoagulation for previous history of pulmonary embolism.

**Table 1:** Maternal characteristics.

Characteristic	Mothers (*N* = 10, corresponding to 14 pregnancies)
At first conception after KTx	
Gestation/parity Previous abortus, *n*/*N* (%) Previous miscarriage, *n*/*N* (%) Pregnancies before KTx, *n*/*N* (%) Time from KTx (months), median (range)^†^ CMV seropositive, *n*/*N* (%) Weight (kg), median (range) ^†^ BMI (kg/m^2^), median (range) ^†^ Comorbidities, *n*/*N* (%) Obesity Chronic hypertension Diabetes mellitus Thrombo-embolism history Fecundation technique, *n*/*N* (%) Artificial insemination IVF	2/10 (20) 2/9 (22) 3/10 (30) 41 (12–112) 4/10 (40) 61.2 (47.3–68.9) 23 (17–26) 0/10 (0) 3/10 (30) 3/10 (30) 3/10 (30) 2/14 (14) 2/14 (14)
Nephrological history RRT, *n*/*N* (%) Duration of dialysis (months), median (range) Ever had PD, *n*/*N* (%) AVF, *n*/*N* (%) Initial nephropathy, *n*/*N* (%) CAKUT Type 1 diabetes Alport syndrome IgA nephropathy Membranoproliferative glomerulonephritis	9/10 (90) 10 (2–144) 3/10 (30) 6/8 (75) 5/10 (50) 2/10 (20) 1/10 (10) 1/10 (10) 1/10 (10)
At last KTx before pregnancy	
Age (years), median (range) Ethnic group, *n*/*N* (%) Caucasian Asian Second transplantation, *n*/*N* (%) Ablation of first graft ablation, *n*/*N* (%) Living donor, *n*/*N* (%) Preemptive transplantation, *n*/*N* (%) Induction therapy *n*/*N* (%) Basiliximab ATG	35 (23–42) 9/10 (90) 1/10 (10) 5/10 (50) 2/5 (40) 7/10 (70) 2/10 (20) 6/9 (67) 3/9 (33)

AVF, arteriovenous fistula; ATG, antithymocyte globulin; BMI, body mass index; CAKUT, congenital anomalies of the kidney and urinary tract; CMV, cytomegalovirus; IVF, *in vitro* fecundation; KTx, kidney transplantation; PD, peritoneal dialysis; RRT, renal replacement therapy.

^†^Patient 8 was excluded because we only have detailed data following her second pregnancy after KTx.

**Table 2:** Obstetrical characteristics.

Characteristic	Pregnancies (*N* = 14)
During pregnancy	
Substance use, *n*/*N* (%) Tobacco Alcohol Medication, *n*/*N* (%) Ciclosporin Tacrolimus Azathioprine Corticosteroids Aspirin Anticoagulants Anti-D antibodies Complications, *n*/*N* (%) Preeclampsia Gestational diabetes Gestational hypertension	1/14 (7) 1/14 (7) 2/14 (14) 12/14 (86) 13/14 (93) 8/14 (57) 1/14 (7) 1/14 (7) 2/13 (15) 2/14 (14) 0/14 (0) 0/14 (0)
Delivery, *n*/*N* (%) Cesarean section Emergency Newborn position, *n*/*N* (%) Breech Head Hemorrhage > 500 mL	11/14 (79) 4/14 (29) 2/13 (15) 11/13 (85) 6/14 (43)
After delivery	
Hospital stay, median days (range)	7 (5–11)

Three patients had already had children before their first KTx. Two of our studied patients had a previous miscarriage after their first KTx (no specific investigation was performed except for antiphospholipid antibodies, which were negative) and two patients had a history of voluntary termination of pregnancy before KTx. One patient admitted using alcohol and tobacco during her pregnancy. Among the 14 live births in our cohort, two (14%) were obtained by *in vitro* fecundation (IVF) and two by insemination (including one artificial insemination after sperm donation). In all these cases, maternal fertility problems had been documented and, interestingly, all patients belonged to the congenital anomalies of the kidney and urinary tract (CAKUT) initial nephropathy group with a long-lasting history of genitourinary infections before transplantation. The median time between KTx and the first following pregnancy was 41 months (range 12–112 months). Interestingly, 5/10 patients had already had a second KTx by the time of their first pregnancy. Reasons for failure of the first KTx were chronic antibody-mediated rejection (20%), severe acute cellular rejection (40%), relapsing pyelonephritis episodes (20%), and chronic allograft nephropathy mainly due to calcineurin inhibitor (CNI) toxicity (20%).

Two patients had a preemptive KTx. For the remaining patients, the median overall time on dialysis (mainly hemodialysis) before KTx was 10 months (range 2–144 months). Seven patients (70%) received their last KTx from a living donor and three (30%) from a deceased donor. One patient had a combined simultaneous pancreas–kidney transplantation (SPK) for type 1 diabetes (Patient 9). Initial nephropathies were CAKUT (50%), type 1 diabetes (20%), Alport syndrome (10%), IgA nephropathy (10%), and membranoproliferative glomerulonephritis (10%). The induction therapy at the last KTx consisted of basiliximab (67%) or antithymocyte globulin (ATG, 33%), with ATG given in the context of a second KTx (Patient 1, Patient 6, and Patient 8). No patient had DSAs at the time of KTx. Patient 10 had a documented histologic recurrence of IgA nephropathy 4 months before her second pregnancy (> 2 years from the first pregnancy), however without significant associated renal dysfunction. Five patients had a history of biopsy-proven acute T-cell-mediated rejection on the current allograft (from borderline to Banff IIb) before conception. Immunosuppressive drugs at conception consisted of triple therapy of tacrolimus (FK), azathioprine (Aza), and prednisone in eight patients (57%), dual therapy of FK and Aza in four patients (29%) or ciclosporin (CsA) and Aza in one patient (7%), and monotherapy with CsA in one patient (7%). During pregnancy, target trough levels of CNI were set slightly higher than in non-pregnant transplant recipients with a similar immunological risk profile (6–8 µg/L and 100–120 µg/L for FK and CsA, respectively). Except for dose adjustments of FK and CsA in accordance with measured trough levels, there were no significant changes in immunosuppressive drugs during pregnancy, with the switch from mycophenolate mofetil (MMF) to Aza taking place before conception. Except for one patient’s therapy, which was modified from FK and Aza to FK and prednisone dual therapy during the first trimester, none received additional immunosuppressive drugs during pregnancy.

### Obstetrical and maternal outcomes

Three patients had more than one live birth pregnancies after KTx: three for Patient 3, and two for Patients 6 and 10. There were no multiple pregnancies, even after IVF treatment. Median gestational time was 36.2 weeks (range 27.3–38.7 weeks). Cesarean section was chosen as the delivery method in 11 pregnancies (79%), including four for different emergency purposes: preeclampsia, decreased fetal movements, fetal bradycardia, and breech. The remaining three pregnancies resulted in a spontaneous vaginal delivery.

Maternal complications during pregnancy comprised the following: stage 1 AKI (43%), urinary tract infections (UTI, 43%), progression of proteinuria without diagnostic criteria for preeclampsia (29%), preeclampsia (14%), hyperemesis gravidarum (7%), severe anemia (7%), oligohydramnios (7%), polyhydramnios (7%), and chickenpox (7%). There were no major obstetrical complications, except that six pregnancies (43%) were complicated by hemorrhage at delivery > 500 mL. Interestingly, in our small cohort, this complication was more frequent in patients with an initial diagnosis of CAKUT (four out of five patients, or four out of six deliveries), than in non-CAKUT patients (two out of five patients, or two out of eight deliveries). Regarding infectious complications, one patient had graft pyelonephritis, and the remaining UTI mainly occurred during the second trimester (70%). Six patients were prescribed antibiotic prophylaxis (cefuroxime or amoxicillin-clavulanate). One patient had post-partum endometritis due to group B *Streptococcus*. None of our patients had a human polyomavirus 1 (BKV) viremia before, during, or at the last follow-up (2 years) after pregnancy. Three women (Patient 3 before her third pregnancy, and Patients 4 and 6) had low-level positive viremia (<2,000 copies/mL) for cytomegalovirus (CMV) ranging from 4 months to 1 year before pregnancy, without increased levels during pregnancy. No specific treatment was introduced except careful monitoring during pregnancy, as all these patients were already CMV seropositive (CMV reactivation). All patients had a positive EBV serology before pregnancy and no specific monitoring was performed.

We did not observe any new onset of arterial hypertension or gestational diabetes in our study. The median systolic and diastolic blood pressures at conception were measured at 116 mmHg (range 91–146 mmHg) and 70 mmHg (range 54–85 mmHg), respectively. None of our patients met the criteria for postpartum preeclampsia, as defined by new onset of preeclampsia between 48 hours to 6 weeks after delivery (21). The two patients who had preeclampsia (Patients 1 and 4) had been diagnosed with chronic arterial hypertension before pregnancy. For Patient 9 (SPK for type 1 diabetes), glucose levels remained controlled throughout pregnancy, without the need for any treatment, demonstrating adequate function of the pancreatic allograft. Her detailed medical history has been reported previously (22). The other type 1 diabetes patient who received KTx alone (Patient 3) experienced poor glycemic controls during all three pregnancies with frequent episodes of hypo- and hyperglycemia, partly explained by hyperemesis and digestive dysmotility.

Out of the seven returned questionnaires about the overall perception of pregnancy after KTx (**Supplementary Figures 1**, **2**), one-third of patients had experienced anxiety during pregnancy, half rather agreed that they were “very preoccupied by the effect of the pregnancy on the allograft”, almost half disagreed that they were “very preoccupied by the effect of the immunosuppressive drugs on the fetus”, and half had considered pregnancy while on RRT, irrespective of KTx. Three patients (43%) could envision another pregnancy. We did not retrieve any information about breastfeeding. However, in recent years we have discouraged breastfeeding in patients under immunosuppressive therapy, in particular CNI.

### Allograft outcome

**Figure 1:**
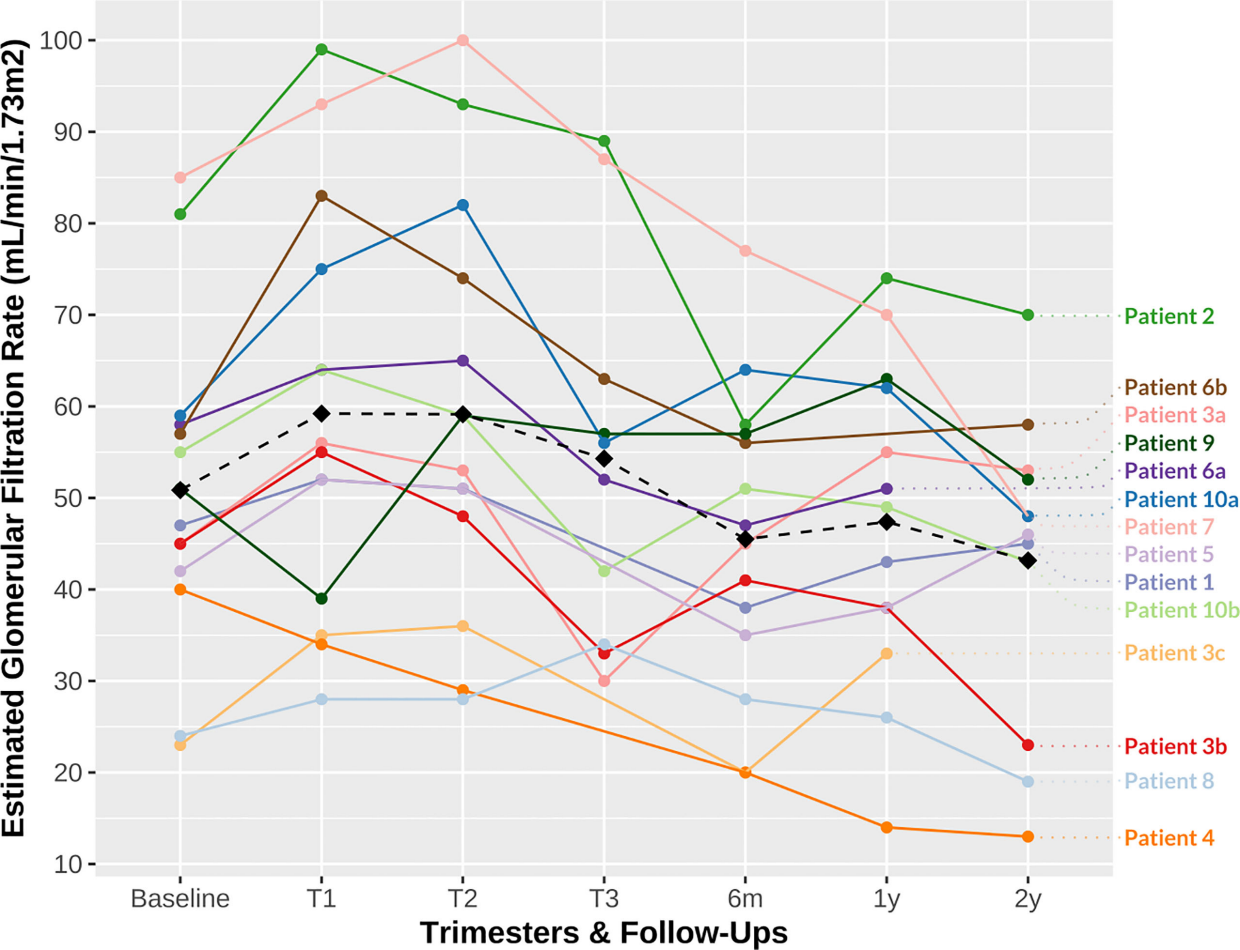
Patient estimated glomerular filtration rate (eGFR) during follow-up. The dashed line represents the mean eGFR (T0 baseline, 50.86 mL/min/1.73m^2^; T1, 59.21 mL/min/1.73m^2^; T2, 59.14 mL/min/1.73m^2^; T3, 54.3 mL/min/1.73m^2^; 6 months, 45.50 mL/min/1.73m^2^; 1 year, 47.38 mL/min/1.73m^2^; 2 years, 43.17 mL/min/1.73m^2^). eGFR, estimated glomerular filtration rate; m, month; T, trimester; y, year. a, first pregnancy; b, second pregnancy; c, third pregnancy.

The median baseline sCr level at conception was 126.5 μmol/L (range 84.0–240.0 µmol/L), corresponding to a median eGFR of 49 mL/min/1.73m^2^ (range 23–85 mL/min/1.73m^2^). One patient had an eGFR at conception of less than 30 mL/min/1.73m^2^, and one patient had a uPCR greater than 30 mg/mmol at conception. As expected, we observed a decrease in median sCr levels during pregnancy, mostly within the first trimester: median sCr levels during the first, second, and third trimesters were 111.5 μmol/L (range 71–207 μmol/L), 114.5 μmol/L (range 73–210 μmol/L), and 120.5 μmol/L (range 77–185 μmol/L), respectively, corresponding to a median eGFR of 55.5 mL/min/1.73m^2^ (range 28–99 mL/min/1.73m^2^), 56 mL/min/1.73m^2^ (range 28–100 mL/min/1.73m^2^), and 54 mL/min/1.73m^2^ (range 30–89 mL/min/1.73m^2^), respectively. However, at the consecutive follow-ups after delivery, eGFR tended to be lower than baseline (**Figure 1**): the median eGFR at 6 months, 1 year, and 2 years was 46 mL/min/1.73m^2^ (range 20–77 mL/min/1.73m^2^), 49 mL/min/1.73m^2^ (range 14–74 mL/min/1.73m^2^), and 47 mL/min/1.73m^2^ (range 13–70 mL/min/1.73m^2^), respectively. Three patients had an eGFR of less than 30 mL/min/1.73m^2^ at 6 months and 2 years after delivery. One patient (Patient 3) underwent an SPK between the 1st and 2nd year of follow-up after her third pregnancy due to progressive allograft dysfunction (those latter graft function values were then noted as “not applicable” for this study).

Anti-HLA antibodies and DSAs were regularly monitored before, during, and after pregnancy. Only one patient (Patient 3) had pre-existing DSAs before her (third) pregnancy: moderate levels of class I (A1 and CW18, with cumulative mean fluorescence intensity, cMFI, < 5,000) and class II (DR7 at 4061 MFI) anti-HLA antibodies, which all became undetectable during and after pregnancy. Overall, although some patients displayed an increase in preexisting or the occurrence of *de novo* anti-HLA antibodies (even at high titers), none were DSAs. This may be related to non-specific immune activation during pregnancy, but no patient had clinical features of an acute rejection episode and no kidney biopsy was performed during gestation. Nevertheless, five patient (50%) had an allograft biopsy during follow-up after delivery due to persisting elevated sCr levels or proteinuria. Two biopsies were done within 6 months, one between 6 months and 1 year, and two between 1 year and 2 years after delivery, showing chronic CNI toxicity (60%) and different stages (borderline to Banff IIb) of acute T-cell-mediated rejection (40%). One patient was treated for clinically suspected acute cellular rejection within the 1- to 2-year follow-up interval without biopsy documentation. There was no documented graft loss within the 2-year follow-up period, but two patients were relisted for a preemptive transplantation (KTx alone and SPK) due to progressive allograft dysfunction following pregnancy.

### Offspring outcomes

The short-term characteristics of the newborns are summarized in **Table 3**. Ten babies were female (71%) and four were male, with a median gestational age of 35.7 weeks (range 27.2–40.5 weeks). There were nine premature births (64%), including two very preterm births (30.6 and 30.7 weeks) and one extremely preterm birth (27.2 weeks), with a median length of stay in neonatal care facilities of 9 days (range 3–48 days). Three of the preterm births were induced. Two newborns had an Apgar score of less than 8 at 1 minute. Neonatal complications were acute respiratory distress syndrome (43%), infant respiratory distress syndrome (21%), hyperbilirubinemia (36%), arterial hypotension (14%), hypoglycemia (14%), and AKI (7%). Mosaic Turner syndrome was diagnosed in one child. Other malformations, *ductus arteriosus* (29%) and *foramen ovale* (14%), were essentially on the cardiothoracic level. We did not find any renal malformations.

**Table 3:** In-hospital newborn characteristics.

Characteristic	Newborns (*N* = 14 from 10 mothers)
Before delivery	
Normal amniotic index, *n*/*N* (%) Gestational age (weeks), median (range)	10/14 (83) 36.2 (27.3–38.7)
Gender, *n*/*N* (%) Female Male	10/14 (71) 4/14 (29)
Measurements SGA, *n*/*N* (%) Weight (g), median (range) Height (cm), median (range) Head circumference (cm), median (range)	2/14 (14) 2,560 (860–3,550) 45.5 (34–51) 32.1 (24.5–34.5)
Neonatal period	
Prematurity, *n*/*N* (%) Neonatology stay (days), median (range) Complications, *n*/*N* (%) ARDS IRDS Hypoglycemia Hypotension Hyperbilirubinemia AKI Malformations, *n*/*N* (%) Cardiac Ductus arteriosus Renal	9/14 (64) 9 (3–48) 6/14 (43) 3/14 (21) 2/14 (14) 2/14 (14) 5/14 (36) 1/14 (7) 2/14 (14) 4/14 (29) 0/14 (0)

ARDS, acute respiratory distress syndrome; AKI, acute kidney injury; IRDS, infantile respiratory distress syndrome; SGA, small for gestational age.

**Figure 2:**
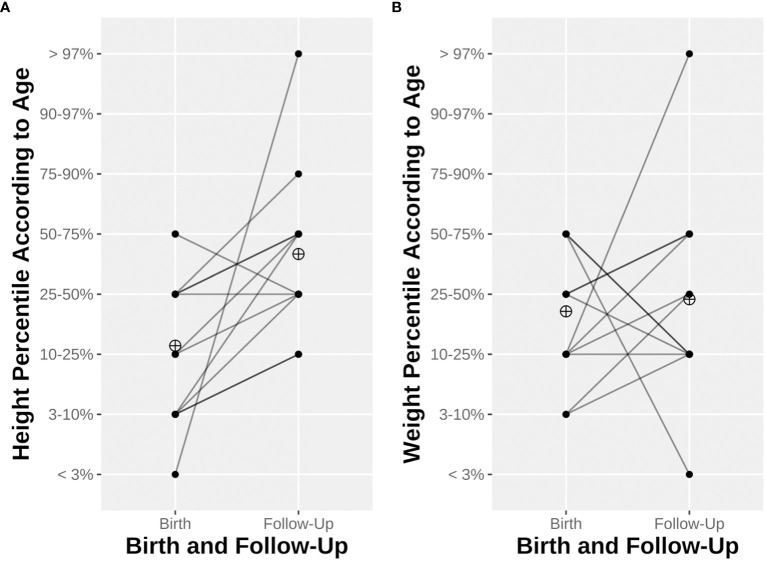
Child height **(A)** and weight **(B)** at birth and until last follow-up. Line opacity depends on the number of observations. The ringed cross represents mean height percentile **(A)** or weight percentile **(B)**.

The median birth weight, height, and head circumference were 2,560 g (range 860–3,550 g), 45.5 cm (range 34–51 cm), and 32.1 cm (range 24.5–34.5 cm), respectively. Two (14%) infants were SGA. Furthermore, 10 (71%), 12 (86%), and 10 out of 12 (83%) had a weight, height, and head circumference under the 50th percentile, respectively, of whom two (14%), five (35%), and two (17%), respectively, were even under the 10th percentile for these measurements. We obtained follow-up measurements for 12 out of 14 children *via* the questionnaires sent to their mothers at the time of the study. The median age at follow-up was 11.5 years (range 4–18 years). The progression of the children’s height and weight according to percentiles for age is displayed in **Figure 2**. Seven (58%) of the children reached at least the next percentile in terms of weight, and ten (71%) did so in terms of height. Conversely, four (33%) had a worse development reaching lower percentiles in terms of weight and only one (8%) in terms of height. The median BMI according to age was between the 25th and the 50th percentiles. None had been hospitalized, and two were prescribed one medication (i.e., iron and levothyroxine). One child had been diagnosed during follow-up with abdominal aortic aneurysm and bicuspid aortic valve regurgitation; no other pathologies were noted. Their overall health, psychological health, social skills, and education at follow-up were all rated relatively high scores (> 7 out of a maximum of 10) by their mothers. The long-term development of the offspring is summarized in **Table 4**.

**Table 4:** Long-term development of the offspring.

Characteristic	Children (*N* = 12 from returned questionnaires)
Median follow-up (years), median (range)	11.5 (4–18)
Medical history, *n*/*N* (%) Need for hospitalization Need for specific medication Renal impairments Other anomalies	0/12 (0) 2/12 (17) 0/12 (0) 1/12 (8)
Mother’s assessment on a scale from 1 (worst) to 10 (best), median (range) Overall health Psychological health Social skills Education	10 (7–10) 8.5 (8–10) 10 (8–10) 9 (7–10)

## Discussion

This observational study summarizes our experience with pregnancies after KTx that took place between 2000 and 2021 in a tertiary hospital in Switzerland. The small number of pregnancies (10 patients with 14 pregnancies) during this time period can be partly explained by the epidemiology of ESRD patients undergoing KTx (mainly men with a median age at KTx of 53.4 years in our center), but also possibly by fears from the patients and the medical teams of complications that could compromise the outcome of the allograft. We have compared our data to data available from the prospective ongoing Swiss Transplant Cohort Study (STCS) (23, 24). Since May 2008 to January 2023, according to the STCS dataset, there were 24 live births among the 1,603 women who have undergone a KTx in Switzerland, of whom 554 (34.6%) were of childbearing age (15–49 years old) at the time of transplantation. Our center thus represents 41.7% of the live birth pregnancies after KTx in our country (4/14 pregnancies presented in this study occurred before 2008 and recruitment by the STCS). The percentage of births among STCS KTx women of childbearing age (0.04%) was clearly below the nearly 3% reported in the Swiss general population in 2021 (25).

### Mother outcome

The mean age at conception was slightly higher in our cohort (33.6 years) than in the overall Swiss population (32.2 years in 2020) (26) and as described in the literature for women after KTx (29.6 years). Globally, KTx women appear to have similar live birth pregnancy rates to the general population, irrespective of geographical region, whereas rates of stillbirth and neonatal mortality remain significantly higher (8). Spontaneous abortions were reported to mostly occur in KTx women aged < 25 year and > 35 years (8), with more live births in patients < 30 years of age (9). In addition, a slightly higher rate of ectopic pregnancies has been reported in KTx women. The retrospective nature of our study precluded a reliable analysis of non-evolutive pregnancies in our patients of childbearing age, as these outcomes were not systematically recorded. Nearly one-third of our patients had several pregnancies after KTx, which is consistent with previous studies (11).

Despite improvements in fertility after KTx, couples may resort to medically-assisted procreation. The first reported case of IVF (and embryo transfer) resulted in a preterm twin delivery, with low birth weights, and was complicated by a deep right iliac vein thrombosis in the mother (27). In non-transplanted women, IVF pregnancies lead to more adverse outcomes (i.e., preterm birth, SGA, and perinatal mortality) than spontaneous conception pregnancies, even if only singleton IVF pregnancies are considered (28). Interestingly, studies focusing solely on KTx women have shown relatively similar and reassuring pregnancy outcomes after IVF in comparison with spontaneous conception (28, 29). Nevertheless, pre-implantation procedures tend to limit the number of re-implanted embryos to favor singleton pregnancies (27). In our cohort, two (14%) of the 14 pregnancies were obtained by IVF (Patients 5 and 6a). Graft outcomes were relatively good for those patients, but Patient 5’s child was born prematurely with low birth measurements (< 10th percentile) and a particularly long stay in neonatal facilities (48 days). All patients waited at least one year after KTx to conceive, but three conceptions occurred less than 2 years after KTx (median time 4.1 years, all pregnancies taken into consideration). The best timing remains uncertain: European guidelines advocate delaying conception until 2 years after KTx, whereas American guidelines recommend an optimal window between 1 and 2 years after KTx (30, 31). The meta-analysis by Deshpande et al. found better pregnancy outcomes but higher rates of preeclampsia, cesarean section, and preterm birth in pregnancies < 2 years from KTx (9). Likewise, Shah et al. showed lower spontaneous and induced abortion rates, and less neonatal deaths, in the < 2-year interval from KTx than in the 2- to 3-year interval, with reduced rates of preeclampsia (8). In our center, we advise postponing pregnancy to 1–2 years after KTx. The rationale is to obtain a steady state in kidney function, metabolic and immunological parameters, and maintenance immunosuppression before conception.

More than half of the patients were under a triple CNI-based immunosuppressive regimen. None had MMF during pregnancy, which was replaced by Aza or discontinued at least 3 months before conception following recommendations (30, 31), given the major risk of miscarriages and congenital anomalies (32). Tacrolimus was the agent of choice, despite the fact that levels may be difficult to adjust during pregnancy and that its endothelial and renal toxicity can contribute to preeclampsia (33). Nearly one-quarter of KTx women develop pregnancy-induced hypertension and 5% develop gestational diabetes (8). We did not observe these rates in our small cohort, with a median BMI at conception that was similar to previous studies (11). In meta-analyses, the presence of chronic arterial hypertension, elevated sCr levels (> 125–132 μmol/L), proteinuria, and previous history of preeclampsia were associated with poorer pregnancy outcomes (e.g., miscarriage, preterm delivery, low birth weight, and cesarean section) (9) and a higher risk of preeclampsia (34). The preeclampsia rate in our cohort (14%) was lower than reported in previous meta-analyses (21.5%–27%) but remained much higher than in the general population (8, 9). Preeclampsia occurred in one-third of the patients in the large cohort studied by Gosselink et al. (11). Importantly, preexisting hypertension or proteinuria may hamper the timely diagnosis of preeclampsia but should be discussed at conception, particularly with older patients (8). Preeclampsia has been documented as a risk factor for cesarean section, preterm delivery, SGA newborns, and graft dysfunction. However, a deleterious effect on long-term eGFR and graft loss was not confirmed in multicenter studies and meta-analyses (6, 35). Regarding preventive measures, only one patient in our cohort was prescribed aspirin at the time of pregnancy. The use of low-dose aspirin, introduced before 16 weeks of gestation, has been demonstrated to reduce the risk of preterm preeclampsia in high-risk patients, while slightly increasing the risk of post-partum hemorrhage and placental abruption (36, 37). This primary prevention has not been studied specifically in KTx women and was not incorporated into previous American and European guidelines (30, 31). Nevertheless, many authors suggest aspirin use in KTx women, because early introduction is generally safe (12, 38, 39). Due to the time frame of our study, our patients could not benefit from the now routinely available monitoring based on biomarkers such as sFlt-1/PIGF (40).

The rate of UTI during pregnancy is rarely discussed in KTx patients, although UTI may carry a risk of preterm birth and SGA infants. This rate in our cohort (43%) was significantly higher than in the general non-transplanted population (1%–2%) (41), but is consistent with the 30%–50% described in pregnant KTx women (42, 43) and highlights the importance of regular screening (12). No patient developed primary or reactivation of viral infections during pregnancy. Vertical transmission of CMV in non-KTx women is much higher during primary infection than reactivation. There are less adverse outcomes on the fetus if maternal CMV infection occurs within the third trimester than if viremia and transmission occur during the first trimester (44). Unfortunately, few articles discuss the specific monitoring of CMV in pregnant KTx women.

KTx patients are at increased risk of cesarean section, which is partially explained by emergency indications. The rate of cesarean section (79%) in our cohort was double that in the overall Swiss population (32.6% in 2020), and even greater than those in previous reports (8, 9, 26). Only one-third of the cesarean sections in our cohort was carried out for emergency purposes. In the literature, delivery is induced in about one-quarter of preterm births (8), but the decision to induce delivery seems to have become more frequent over the years (11). Whether this is a result of clinicians’ uneasiness toward KTx or the genuine prevention of pregnancy complications warrants further study.

### Allograft outcome

Even though some degree of allograft hypertrophy and hyperfiltration already occurs after KTx as a compensatory mechanism, physiological changes enable KTx women to reach better eGFR during pregnancy, providing a baseline preserved nephron mass. The 24-hour creatinine clearance has been demonstrated to increase by about 30% in the first trimester, peaking at week 10 in KTx women, then decrease during the second and third trimesters to reach sCr levels relatively similar to pre-pregnancy levels (45). The increase in eGFR during the first trimester was observed to be lower in grafts with delayed graft function at the time of KTx and in grafts with lower sCr levels at conception (45). Physiological changes in eGFR were observed in our cohort, although these were less pronounced (16% difference between mean eGFR at conception and at first trimester: 50.86 mL/min/1.73m^2^ and 59.21 mL/min/1.73m^2^, respectively; **Figure 1**). The short- and long-term follow-up eGFR were also less favorable for our patients than those in previous reports (6), with a 15% decrease. The small cohort size, the heterogeneity of the mothers’ baseline characteristics (e.g., higher rates of proteinuria at conception) and the multiparity may explain the poorer outcome. Proteinuria was observed to rise up to threefold by the third trimester in KTx pregnant women, with high inter-individual variations (45). Hyperfiltration of the (single) kidney allograft may to some extent underly proteinuria in pregnant KTx women (45). We observed a progression in proteinuria without criteria for preeclampsia in nearly one-third of our cohort.

Acute rejection has a reported occurrence of 4%–9% in KTx women who have a pregnancy, but this rate does not significantly differ from non-pregnant KTx women (8, 9). In our cohort, we did not witness any rejection during pregnancy, but we documented two biopsy-proven and one clinically suspected acute rejection episodes during the 2 years of follow-up (2%). In our cohort, there was neither any increase in preexisting DSAs nor occurrence of *de novo* DSAs. Among sensitizing events (e.g., blood transfusions and re-transplantation), pregnancy is a major cause of anti-HLA-antibody formation, especially class I anti-HLAs (10). The levels of alloantibodies have been associated with parity and linked to higher risks of antibody-mediated rejection in non-pregnant KTx women and possibly preeclampsia (6, 10). However, Gatault et al. showed that women without preexisting anti-HLA antibodies at conception and who had already had a pregnancy before KTx had a significantly lower risk of developing DSAs (46). Overall, the data suggest that pregnancy after KTx has a relatively low risk of adverse immunization outcomes, and these may be related to the father’s HLA (47, 48). Careful immunological monitoring and management of the immunosuppressive drugs before, during, and after pregnancy is therefore important to ensure the best graft outcome.

The incidence of graft loss has been reported to be about one-tenth for KTx women with pregnancies within 2–5 years of delivery and about one-fifth (22.3%) within 5–10 years post partum. This was nevertheless not significantly different from nulliparous KTx women, and graft survival even appeared to be higher than in the overall KTx population (6, 9). This latter effect may be due to a “pre-selection” in those that undertake pregnancy of the best patients and grafts. Median time to graft loss after delivery was 6.44 years in a Dutch cohort (11). Elevated sCr levels (> 132 μmol/L), chronic hypertension, and proteinuria (> 1g/24 hours) at conception were associated with graft loss (some within 2 years after pregnancy) (6, 9). Conversely, the type of delivery, type of donor, and immunosuppressive regimen did not significantly determine graft loss (6). An elevated sCr level of > 150 μmol/L before pregnancy was linked to higher postpartum sCr levels in one study (49), but this difference between pre- and post-partum sCr was no longer found at later follow-ups (> 2 years) (6). Five of our patients had sCr levels > 132 μmol/L at conception and the mean baseline eGFR was < 60 mL/min/1.73 m^2^ (CKD stage 3). This is consistent with the known average kidney function after KTx and with the Dutch cohort described by Gosselink et al. (11). Whereas general guidelines recommend an eGFR > 60 mL/min/1.73 m^2^, we nevertheless support KTx women in their path to motherhood (3, 12). We did not observe any graft loss during the 2-year follow-up, but two patients were relisted in that time frame due to progressive renal dysfunction following pregnancy. Details about pregnancy outcomes in SPK or other combined transplantations are limited to small case series. Hypertension, preeclampsia, cesarean section, and preterm birth seem common in SPK recipients (50–52). The only SPK recipient in our cohort (Patient 9) had a progression of proteinuria and delivered a preterm baby, however without onset of hypertension (22).

The need for monitoring of BKV viremia during pregnancy is unclear, and literature on graft and fetal outcomes is almost non-existent, although pregnancy has been demonstrated as a trigger for BKV reactivation and vertical transmission is possible (53). Only one case of successful pregnancy in a KTx women with a history of polyomavirus nephropathy has been reported, in 2007, but conception happened in the context of a second KTx and prolonged negative BKV viremia (> 3.5 years) (54). None of our patients had a history of BKV viremia or nephropathy.

### Offspring outcome

Despite a trend toward lower pre-pregnancy eGFR values in KTx women in the past decade, the incidences of prematurity and low birth weight do not seem to differ significantly (11). The rate of preterm birth in our cohort is higher than in previous meta-analyses, but with a relatively consistent mean gestational age (35.0 weeks) and low birth weight (2,377 g) (8, 9, 11). The rate of prematurity and the mean birth weight in the Swiss general population in 2021 was 6.4% and 3,308 g, respectively (55). Only 14% of the newborns in our cohort were SGA, which is less than half of previously documented proportions. The use of a national chart was shown to better estimate the risk of mortality in newborns, informing our decision to use a validated national growth chart instead of an international one to classify newborns according to percentiles (56). The growth progression of KTx offspring appears to be normal, but with a higher risk of obesity due to CNI exposure (13). All studied children in our cohort remained under the 50th percentile at follow-up, but only 3/12 (25%) and 1/12 (8%) were under the 10th percentile in terms of BMI and weight, respectively. None was under the 10th percentile for height. One-third of the offspring experienced a weight drop to a lower percentile, but the rest of the children seemed to at least remain on their curves. These data are reassuring, but little extrapolation can be performed. In addition to the small size and the age heterogeneity of the offspring cohort, we could not retrieve all parents’ measurements to determine the theoretical growth curve of the children, and thus could not assess their potential ability to catch up.

None of the newborns had an Apgar score < 5 at 5 minutes after birth, compared with the reported 8% in a Dutch cohort (11). Prematurity and low birth weight have been linked to a higher risk of neonatal AKI, and the long-term development of CKD and other adverse kidney outcomes (57). CNI exposure may also promote neonatal AKI and electrolyte disorders. Only one newborn suffered from AKI during neonatal care. Fortunately, no renal malformation or dysfunction was observed in later follow-ups. The absence of renal and neuro-behavioral impairment in our cohort is consistent with previous studies (13). Children of KTx women were described to be at increased risk of malformation, especially cardiovascular defects, without survival implications in the first year after birth, and hospitalization for infections is also greater in KTx offspring than in the general population (58). Exposure to chronic hypertension and to the mother’s immunosuppressive therapy have been implicated in these outcomes, respectively. Overall, transparency about the paucity of evidence is of utmost importance when advising parents about the long-term development of KTx patients’ offspring (38). This highlights the need for detailed prospective recording of the child’s history (e.g., growth, renal function, infections, and neuro-behavioral development).

## Conclusions and perspectives

This observational study details our experience of pregnancies after KTx in a Swiss tertiary hospital in the last 20 years. Those pregnancies remained at high risk for the mother, with a high prevalence of cesarean sections, emergency deliveries, UTI, and preeclampsia, and for the child, with a high proportion of prematurity and associated complications, lower measurements at birth, and a tendency to stay under the 50th percentile but above the 10th percentile in growth charts during childhood and adolescence. There was also an impact on the allograft, with a trend toward lower eGFR at 2 years after pregnancy. We thus highlight the risks associated with pregnancy after KTx that need to be discussed with patients before conception and the challenges faced by medical teams during short- and long-term follow-up. We also provide some data that are scant in the literature, in particular regarding offspring outcomes beyond the neonatal period.

Our study has several limitations. Above all, this is a single-center retrospective study without matched control groups of non-pregnant KTx and pregnant non-KTx women, hampering causality conclusions. The selection of such matched groups would have been complex. Moreover, we reported on a small cohort with heterogeneous patient characteristics, precluding analyses about potential predictive factors of poor pregnancy outcomes. We also included in our results several pregnancies from the same patients. Mean, median, and range values were thus more influenced by these patients. Many features of our cohort could carry a reporting or a selection bias toward relatively healthier KTx women or pregnancies, with subsequent better outcomes. First, we only included patients who had at least one live birth, potentially overlooking women who exclusively suffered from non-favorable pregnancies. Second, a majority of our patients had a living donor, although it seems that the donor type does not drive pregnancy outcomes (59). Third, our small cohort contains several women with iterative pregnancies. This has undoubtedly singled out patients with encouraging outcomes, more regular or strict gynecological follow-ups, and with possible higher medical literacy and specialist trust. Fourth, initial nephropathies were not distributed as in usual cohorts, with most of our patients suffering from non-recurrent and non-immunological renal diseases. Finally, we have an upstream selection of lower risk KTx women before conception, as in our center we strongly discourage pregnancy in patients with risk factors such as advanced age, severe graft dysfunction (sCr levels > 180–200 μmol/L), uncontrolled arterial hypertension or proteinuria (> 1–2 g/24 h), a recent episode of cellular or antibody-mediated rejection (< 1 year), or uncontrolled recurrence of the initial nephropathy.

Overall, we support Jesudason and Piccoli, who advocate for a large international dataset, which will have to incorporate comprehensive clinical data and monitoring modalities during pregnancy (taking into account the mother, allograft and fetal outcomes, as discussed), and relevant information regarding non-evolutive pregnancies (i.e., early miscarriages and terminations) (3). This might provide more evidence for preventive measures, the stratification of risk factors, management during pregnancy and follow-up, and counseling for parents (38). Moreover, the exposure to immunosuppressive therapies during pregnancy could have a remanent impact. A standardized and longer follow-up with extensive information about children’s outcomes in large cohort studies is needed (13, 14). Lastly, outcome disparities across countries warrant further research to reduce inequalities and to implement region-specific guidelines.

## Data availability statement

The original contributions presented in the study are included in the article/**Supplementary Material**. Further inquiries can be directed to the corresponding author.

## Ethics statement

The studies involving human participants were reviewed and approved by the institution’s Ethics Committee (CER-VD). The patients/participants provided their written informed consent to participate in this study.

## Author contributions

LS, CV, YV, and DG collected and analyzed the data. LS and DG prepared the figures/tables and drafted the manuscript. J-PV, DB, HL, YV, and DG contributed to the follow-up of the patients. All authors reviewed the drafted and final submitted manuscript. All authors contributed to the article and approved the submitted version.

## Supplementary material

Supplementary Figure 1 Questionnaire distributed to the mothers. English translation of the original French version.

Supplementary Figure 2 Summary of the patients’ perception of pregnancy. Sub-analysis of the filled questionnaires (**Supplementary Figure 1**).
