# Supplementary material for: Pregnancy after kidney transplantation: an observational study on maternal, graft and offspring outcomes in view of current literature
Source: Front Nephrol. 2023 Jul 27;3:1216762. doi: 10.3389/fneph.2023.1216762 (PMC10479688; doi:10.3389/fneph.2023.1216762)
Supplement: Supplementary Figure 1 — Questionnaire distributed to the mothers. English translation of the original French version. [file DataSheet_1.pdf]

Questionnaire: Pregnancy after kidney transplantation and children outcomes

This questionnaire is intended for all female patients who have experienced a pregnancy after a kidney transplant. It is part of a study conducted on the outcomes of the mother, the child, and the kidney graft after pregnancy. We kindly ask you to answer all the questions as honestly as possible. Participation in this study is anonymous.

General information

Name

Surname

Date of birth

Nationality

Place of residence

Civil status

Household type ☐ Single parent ☐ Couple ☐ With a first-degree relative ☐ With an unrelated person

Employment status ☐ Employed ☐ Job-seeking ☐ In training ☐ Incapacitated ☐ Stay-at-home

Highest level of education ☐ Compulsory education ☐ Middle school ☐ Higher education, University

Years in dialysis before kidney transplantation (start date if known)

Are you currently in menopause? If yes, since when?

Do you currently use any contraceptive method? If yes, which one?

Pregnancy(ies) before your 1st kidney transplantation

Have you been pregnant before the transplant? If yes, in which year(s)?	
Did you use assisted reproductive techniques before the transplant?	
Have you experienced any miscarriages before the transplant? If yes, in which year(s)?	
Have you had any voluntary terminations of pregnancy before the transplant? If yes, in which year(s)?	

Pregnancy(ies) after your 1st kidney transplantation

How many times have you been pregnant after the transplant?	
Did you use assisted reproductive techniques after the transplant?	
Did you discuss with your doctor at the transplantation center before considering a pregnancy?	
Have you experienced any miscarriages after the transplant? If yes, in which year(s)?	
Have you had any voluntary terminations of pregnancy after the transplant? If yes, in which year(s)?	
Do you still have gynecological follow-up? If yes, how often?	
Did you smoke during your pregnancy? Did you consume alcohol during your pregnancy?	

For the following statements, please indicate the option that best corresponds to your first pregnancy after the 1st transplant.

	Completely agree	Agree	Neutral	Disagree	Completely disagree
My pregnancy went smoothly without any complications.	O	O	O	O	O
I was particularly anxious during this pregnancy.	O	O	O	O	O
I was very concerned about the effects of medication on the baby.	O	O	O	O	O
I was very concerned about the effect of pregnancy on the graft.	O	O	O	O	O
I would not have considered this new pregnancy if I had not undergone transplantation.	O	O	O	O	O
I am considering another pregnancy/I would be ready for another pregnancy.	O	O	O	O	O

Children BEFORE the first kidney transplantation

How many children do you have in total? ______ of whom ______ before and ______ after transplantation.

Please provide the following information for each of the children you had before the first kidney transplant.

Child 1

Current age

Current weight/height

Current level of education

Usual medication intake? If yes, which one(s)?

Required hospitalization? If yes, for what reason(s)?

Experienced any kidney-related issues? If yes, which ones?

On a scale of 1 to 10 (1 being the lowest, 10 being the highest), please estimate for your child:

Overall health condition	1	2	3	4	5	6	7	8	9	10
Mental health	1	2	3	4	5	6	7	8	9	10
Sociability	1	2	3	4	5	6	7	8	9	10
Academic performance	1	2	3	4	5	6	7	8	9	10

Children AFTER the first kidney transplantation

Please provide the following information for each of the children you had after the first kidney transplant.

Child 1

Current age

Current weight/height

Current level of education

Usual medication intake? If yes, which one(s)?

Required hospitalization? If yes, for what reason(s)?

Experienced any kidney-related issues? If yes, which ones?

On a scale of 1 to 10 (1 being the lowest, 10 being the highest), please estimate for your child:

Overall health condition	1	2	3	4	5	6	7	8	9	10
Mental health	1	2	3	4	5	6	7	8	9	10
Sociability	1	2	3	4	5	6	7	8	9	10
Academic performance	1	2	3	4	5	6	7	8	9	10

Child 2

Current age

Current weight/height

Current level of education

Usual medication intake? If yes, which one(s)?

Required hospitalization? If yes, for what reason(s)?

Experienced any kidney-related issues? If yes, which ones?

On a scale of 1 to 10 (1 being the lowest, 10 being the highest), please estimate for your child:

Overall health condition	1	2	3	4	5	6	7	8	9	10
Mental health	1	2	3	4	5	6	7	8	9	10
Sociability	1	2	3	4	5	6	7	8	9	10
Academic performance	1	2	3	4	5	6	7	8	9	10

Child 3

Current age

Current weight/height

Current level of education

Usual medication intake? If yes, which one(s)?

Required hospitalization? If yes, for what reason(s)?

Experienced any kidney-related issues? If yes, which ones?

On a scale of 1 to 10 (1 being the lowest, 10 being the highest), please estimate for your child:

Overall health condition	1	2	3	4	5	6	7	8	9	10
Mental health	1	2	3	4	5	6	7	8	9	10
Sociability	1	2	3	4	5	6	7	8	9	10
Academic performance	1	2	3	4	5	6	7	8	9	10

Any comments about the questionnaire
