# Supplementary material for: Pregnancy after kidney transplantation: an observational study on maternal, graft and offspring outcomes in view of current literature
Source: Front Nephrol. 2023 Jul 27;3:1216762. doi: 10.3389/fneph.2023.1216762 (PMC10479688; doi:10.3389/fneph.2023.1216762)
Supplement: Supplementary Figure 2 — Summary of the patients’ perception of pregnancy. Sub-analysis of the filled questionnaires ( Supplementary Figure 1 ). [file DataSheet_2.pdf]

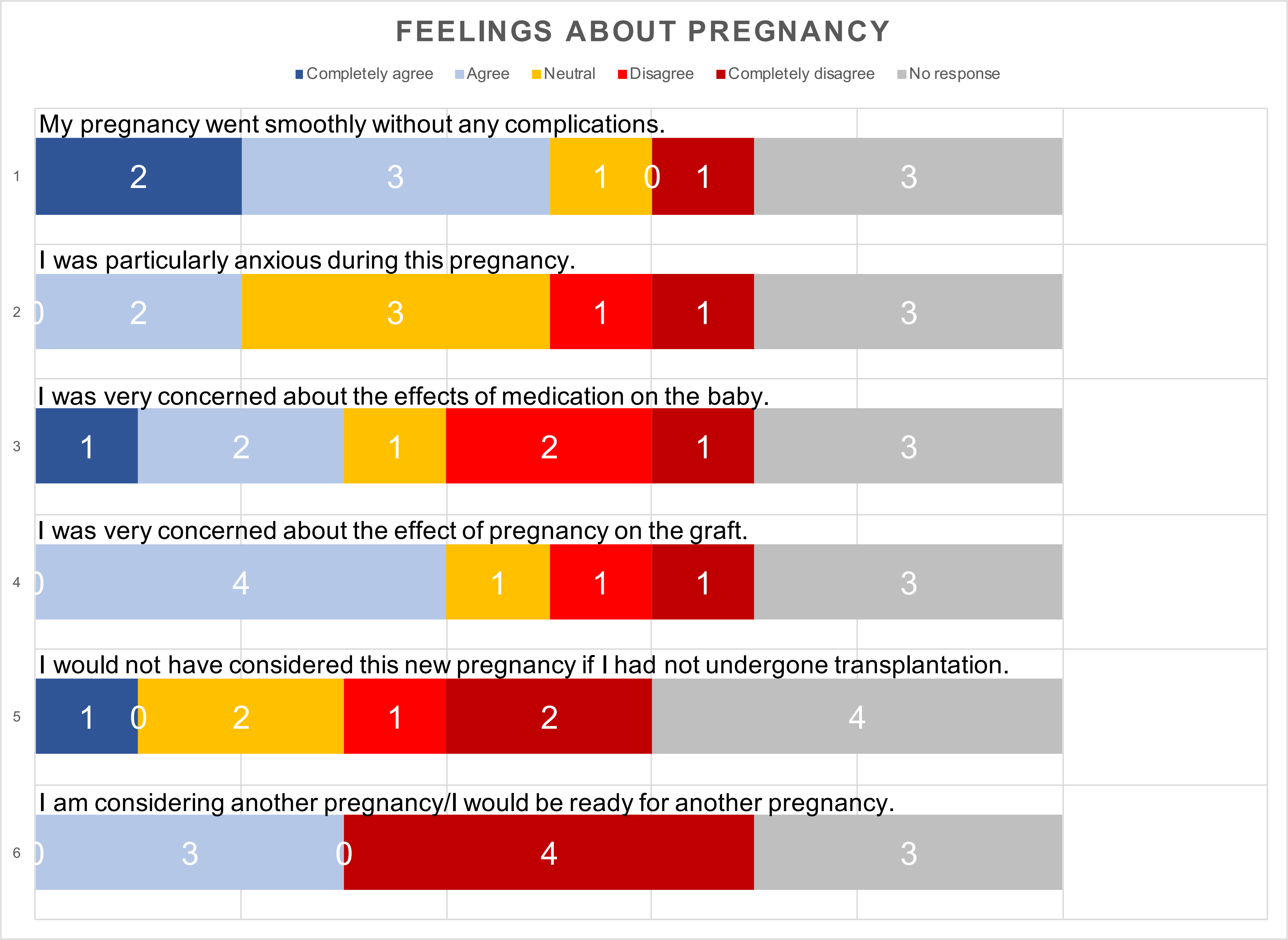
FEELINGS ABOUT PREGNANCY
Completely agree
Agree
Neutral
Disagree
Completely disagree
No response
My pregnancy went smoothly without any complications.
I was particularly anxious during this pregnancy.
I was very concerned about the effects of medication on the baby.
I was very concerned about the effect of pregnancy on the graft.
I would not have considered this new pregnancy if I had not undergone transplantation.
I am considering another pregnancy/I would be ready for another pregnancy.
1 2 3 4 5 6
2 3 1 0 1 3
2 3 1 1 3
1 2 1 2 1 3
4 1 1 1 3
1 0 2 1 2 4
3 0 4 3
